# The anti-inflammatory role of tissue inhibitor of metalloproteinase-2 in lipopolysaccharide-stimulated microglia

**DOI:** 10.1186/1742-2094-11-116

**Published:** 2014-06-27

**Authors:** Eun-Jung Lee, Hee-Sun Kim

**Affiliations:** 1 Department of Molecular Medicine and Global Top5 Research Program, Tissue Injury Defense Research Center, School of Medicine, Ewha Womans University, Mok-6-dong 911-1, Yangchun-Ku, 158-710 Seoul, South Korea

**Keywords:** TIMP-2, MMP, microglia, neuroinflammation, neuroprotection

## Abstract

**Background:**

Tissue inhibitors of metalloproteinases (TIMPs) are known to be endogenous inhibitors of matrix metalloproteinases (MMPs). Our preliminary study showed that TIMP-2 is constitutively expressed in microglia but significantly inhibited by lipopolysaccharide (LPS) treatment. The current study was undertaken to investigate the role of TIMP-2 in microglia.

**Methods:**

The expression of TIMP-2 was evaluated in the BV2 mouse microglial cell line and rat primary cultured microglia. To investigate the role of TIMP-2, a TIMP-2 expression plasmid or small interfering RNA (siRNA) was introduced into BV2 cells by transient transfection, and their effects on LPS-induced inflammatory reactions were examined. We further analyzed the molecular mechanism underlying the anti-inflammatory effects of TIMP-2 by electrophoretic mobility shift assay (EMSA), a reporter gene assay and Western blot analysis.

**Results:**

Overexpression of TIMP-2 significantly inhibited the production of nitric oxide (NO), TNF-α, IL-1β, and reactive oxygen species (ROS), while increasing anti-inflammatory IL-10 production. On the other hand, knockdown of TIMP-2 augmented the production of pro-inflammatory molecules and downregulated IL-10 in LPS-stimulated BV2 cells. The results suggest that endogenously expressed TIMP-2 plays an anti-inflammatory role. Further mechanistic studies revealed that overexpression of TIMP-2 suppresses microglial activation via inhibition of the activity of mitogen-activated protein kinases (MAPKs) and NF-κB with enhancement of the activity of anti-inflammatory Nrf2 and cAMP-response element binding protein (CREB) transcription factors. TIMP-2 also inhibited the activity and expression of LPS-induced MMP-3, -8, and -9. Finally, we demonstrated that TIMP-2 exerts a neuroprotective effect via the inhibition of microglial activation.

**Conclusions:**

Enhancement of TIMP-2 expression may be a potential therapeutic target for neuroinflammatory disorders.

## Background

Microglia, resident immune cells of the central nervous system (CNS), play an important role in brain physiology, including sensing and regulating neuronal activities in the intact brain [1,2]. However, microglia are activated in response to injury and neurotoxic stimuli that can activate inflammatory intracellular signaling pathways, contributing to the release of various proinflammatory/neurotoxic molecules, including nitric oxide (NO), reactive oxygen species (ROS), and cytokines [3-6]. Previous studies have reported that matrix metalloproteinases (MMPs) are also upregulated in activated microglia and play an important role in the pathogenesis of neuroinflammatory disorders [7-9].

Generally, MMP activity is regulated by several mechanisms, including gene transcription, proenzyme activation and inhibition by various endogenous inhibitors such as tissue inhibitors of metalloproteinases (TIMPs) [10,11]. TIMPs bind to active and alternative sites of activated MMPs and inhibit MMP activity by forming a non-covalent complex at a 1:1 molecular ratio. Currently, four different types of TIMPs have been characterized, designated TIMP-1, -2, -3 and -4 [12]. TIMP-2 is constitutively and widely expressed throughout the body, whereas the expression of TIMP-1, -3, and -4 is inducible and often exhibits tissue specificity [10,13].

TIMP-2 is a soluble, non-glycosylated, secreted protein consisting of 194 amino acids. In the adult central nervous system (CNS), the expression of TIMP-2 is enriched with populations of neuronal progenitor cells, suggesting the role of TIMP-2 in neurogenesis [10]. TIMP-2 is also known to be associated with various neuropathological conditions. The TIMP-2 level was reduced in the plasma of patients with frontotemporal dementia [14]. Conversely, TIMP-2 was significantly increased in the serum or cerebrospinal fluid of multiple sclerosis (MS), stroke, Alzheimer’s disease (AD), and Huntington’s disease (HD) patients, suggesting a protective role for TIMP-2 in these diseases [15-17]. Viral vector-mediated delivery of TIMP-2 has been shown to inhibit the development of experimental autoimmune encephalomyelitis or ischemic brain injury [18,19]. In addition, our previous study has proven that TIMP-2 has neuroprotective effects in dopaminergic cell death [20]. Therefore, TIMP-2 has been suggested to be a therapeutic candidate for CNS disorders, such as MS, stroke, and Parkinson’s disease.

Although TIMP-2 is most abundantly expressed in the adult CNS, the function of TIMP-2 in microglia has not been demonstrated until now. We recently reported that TIMP-2 is constitutively expressed in BV2 cells, which is decreased after LPS treatment [8]. However, other TIMP types, such as TIMP-1, -3, and -4, were not detected in BV2 cells, and their expression levels were not altered by LPS treatment. Based on these preliminary findings, we confirmed the mRNA and protein expression of TIMP-2 in BV2 cells and in primary microglia, and analyzed the TIMP-2 function. Using TIMP-2 overexpression and knockdown experiments, we demonstrated, for the first time, that TIMP-2 plays an anti-inflammatory role in LPS-stimulated microglia. Furthermore, we analyzed the detailed molecular mechanisms underlying the anti-inflammatory effects of TIMP-2 in microglia.

## Materials and methods

### Reagents and antibodies

LPS (*Escherichia coli* serotype 055:B5) was obtained from Sigma-Aldrich (St. Louis, MO, USA). All reagents used for cell culture were purchased from Gibco BRL (Grand Island, NY, USA). All reagents and enzymes for reverse transcription polymerase chain reaction (RT-PCR) or oligonucleotides for electrophoretic mobility shift assay (EMSA) were purchased from Promega (Madison, WI, USA). Antibodies against phospho-/total forms of mitogen-activated protein kinases (MAPKs), β-actin or TIMP-2 were supplied by Cell Signaling Technology (Beverley, CA, USA) or Abcam (Cambridge, UK). All other chemicals were obtained from Sigma-Aldrich, unless otherwise stated.

### Microglial and neuronal cell cultures

The immortalized murine BV2 microglial cells and Neuro-2a mouse neuroblastoma cell lines were grown and maintained in Dulbecco’s modified Eagle’s medium (DMEM) supplemented with 10% heat-inactivated fetal bovine serum, streptomycin (10 μg/ml) and penicillin (10 U/ml) at 37°C. Primary microglial cells were cultured from the cerebral cortices of 1- to 2-day-old Sprague-Dawley rat pups as described previously [21]. All animal experiments were approved by the Institutional Animal Care and Use Committee at the School of Medicine, Ewha Womans University. The purity of microglial cultures was greater than 95%, which was confirmed by western blot and immunocytochemisty analyses using an antibody specific to ionized calcium-binding adapter protein-1 (IBA-1; data not shown).

### Tissue inhibitor of metalloproteinase-2 expression plasmid

The control vector (MFG/eGFP/Puro) and TIMP-2 expression vector (MFG/TIMP-2/Puro) were from Dr. Y. S. Kim (Inje University, Seoul, Korea). The MFG/TIMP-2/Puro vector contains the human TIMP-2 cDNA sequence under the control of the viral 5′-LTR sequence and a selection gene (puromycin) [22].

### Transient transfection and luciferase assay

For transient overexpression of TIMP-2, BV2 cells (0.5 × 10^5^ cells per well in a 12-well plate) were transfected with 1 μg of plasmid DNA (control or TIMP-2 plasmid) using the Convoy™ Platinum transfection reagent (CellTAGen, Seoul, Korea). For the nuclear factor kappa B (NF-κB), antioxidant response element (ARE) or cAMP response element (CRE) reporter gene assay, BV2 cells (2 × 10^5^ cells/well in a 12-well plate) were co-transfected with 1 μg of the indicated reporter construct and expression vector (control or TIMP-2 plasmid) mixed with the transfection reagent. After 36 h, cells were treated with LPS (100 ng/ml) and incubated for 6 h prior to harvesting cells. A luciferase assay was performed to determine the effect of TIMP-2 on NF-κB, ARE or CRE promoter activity.

### Reverse-transcription polymerase chain reaction

To monitor gene transcript levels, BV2 cells (7.5 × 10^5^ cells on a 6-cm dish) and rat primary microglial cells (7 × 10^6^ cells on a 6-cm dish) were treated with LPS, and total RNA was extracted with TRIzol reagent (Ambion, Austin, TX, USA). For RT-PCR, total RNA (1 μg) was reverse transcribed in a reaction mixture containing 1 U of RNase inhibitor, 500 ng of random primers, 3 mM MgCl_2_, 0.5 mM dNTPs and 10 U of reverse transcriptase (Promega). The synthesized cDNA was used as a template for PCR using GoTaq polymerase (Promega) and primers as shown in Table 1.

**Table 1:** Primers used in reverse-transcription polymerase chain reaction (RT-PCR) reactions

**Species**	**Gene**	**Forward primer (5′ → 3′)**	**Reverse primer (5′ → 3′)**	**Size**
Mouse	**TNF-α**	CCTATGTCTCAGCCTCTTCT	CCTGGTATGAGATAGCAAAT	354 bp
	**iNOS**	CAAGAGTTTGACCAGAGGACC	TGGAACCACTCGTACTTGGGA	450 bp
	**IL-1β**	GGCAACTGTTCCTGAACTCAACTG	CCATTGAGGTGGAGAGCTTTCAGC	447 bp
	**IL-6**	CCACTTCACAAGTCGGAGGCTT	CCAGCTTATCTGTTAGGAGA	395 bp
	**IL-10**	GCCAGTACAGCCGGGAAGACAATA	GCCTTGTAGACACCTTGGTCTT	409 bp
	**MMP-3**	ATTCAGTCCCTCTATGGA	CTCCAGTATTTGTCCTCTAC	375 bp
	**MMP-8**	CCAAGGAGTGTCCAAGCCAT	CCTGCAGGAAAACTGCATCG	180 bp
	**MMP-9**	GTGATCCCCACTTACTATGGAAAC	GAAGCCATACAGTTTATCCTGGTC	352 bp
	**TIMP-2**	TCTAATTGCAGGAAAGGCAGA	TGCTCTTCTCTGTGACCCAGT	218 bp
	**GAPDH**	ATGTACGTAGCCATCCAGGC	AGGAAGGAAGGCTGGAAGAG	420 bp
Rat	**TIMP-2**	CGTAGTGATCAGAGCCAAGC	TCTGCCTTTCCTGCAATTAGA	225 bp
	**GAPDH**	GTGCTGAGTATGTCGTGGAGTCT	ACAGTCTTCTGAGTGGCAGTGA	292 bp

### Measurement of cytokine and nitrite levels

BV2 cells were treated with LPS (100 ng/ml) for 16 h, and the supernatants were collected. The concentration of TNF-α, IL-1β, IL-6, and IL-10 was measured by enzyme-linked immunosorbent assay according to the manufacturer’s instructions (BD PharMingen, San Diego, CA, USA). Accumulated nitrite was measured using the Griess reagent (Promega).

### Intracellular reactive oxygen species measurement

Intracellular accumulation of ROS was measured using dichlorodihydro-fluorescein diacetate (H_2_DCF-DA; Sigma-Aldrich) as described previously with modifications [23]. Briefly, microglial cells were stimulated with LPS for 16 h and stained with 50 μM H_2_DCF-DA in phosphate-buffered saline buffer for 1 h at 37°C. DCF fluorescence intensity was measured at a 485-nm excitation and a 535-nm emission on a fluorescence plate reader (Molecular Devices, Sunnyvale, CA, USA). Data are presented as fold changes of control-treated values.

### Knockdown of tissue inhibitor of metalloproteinase-2 by small interfering RNA

BV2 cells (0.5 × 10^5^ cells per well in a 12-well plate) were transiently transfected with 100 pM of TIMP-2 small interfering RNA (siRNA) using Ambion siPORT™ NeoFX™ transfection reagent following the manufacturer’s protocols (Ambion). A scrambled control siRNA was used as a negative control. The siRNA sequences are as follows: TIMP-2 siRNA #1 (5′-GAAGGAGUAUCUAAUUGCATT-3′), TIMP-2 siRNA #2 (5′-UCCACAGACUUCAGCGAAU-3′), and Control siRNA (5′-AAUCGCAUAGCGUAUGCCGUU-3’). The cells were harvested 48 h after siRNA transfection, and the expression levels of TIMP-2 protein were measured by western blotting.

### Assays for matrix metalloproteinase-3, -8, and -9 activities

BV2 cells transfected with the TIMP-2 expression plasmid or siRNA were treated with LPS for 24 h, and the supernatants were collected to measure MMP activity using the SensoLyte™ 520 MMP assay system (AnaSpec, San Jose, CA, USA). MMP activity measurements were performed by continuous detection of peptide cleavage using a fluorescence plate reader (Molecular Devices). MMP activity units were expressed as a change in the fluorescence intensity at an excitation of 490 nm and an emission of 520 nm.

### Electrophoretic mobility shift assay

Nuclear extracts from treated microglia were prepared as described previously [24]. The double-stranded DNA oligonucleotides containing NF-κB, ARE, and CRE consensus sequences (Promega) were end-labeled using T4 polynucleotide kinase (New England Biolabs, Beverly, MA, USA) in the presence of [γ-^32^P] ATP. Five micrograms of the nuclear proteins were incubated with ^32^P-labeled probes on ice for 30 min and were resolved on a 5% acrylamide gel as previously described [24]. For competitive binding assays, binding reaction reagents and nuclear extracts were mixed with nonradioactive oligonucleotides in molar excess and incubated before adding the ^32^P-labeled probe.

### Western blot analysis

To detect MAPK activation and TIMP-2 expression, cells lysates were prepared as previously described [24]. To detect secreted TIMP-2, TIMP-2 protein in the conditioned media was enriched using an Amicon™ centrifugal filter (Millipore Corp., Billerica, MA, USA). Proteins were separated on a 12% SDS-polyacrylamide gel and transferred to nitrocellulose membranes (GE Healthcare, Chalfont St. Giles, Buckinghamshire, UK). After blocking of the membranes with 5% skimmed milk in Tris-buffered saline with Tween 20 (TBST), the membranes were incubated with primary antibodies (1:1000) and then were incubated with horseradish peroxidase-conjugated secondary antibodies (1:2000 dilution in TBST; New England Biolabs, Beverly, MA, USA), and the blots were developed using an enhanced chemiluminescence detection kit (Thermo Fisher Scientific, Waltham, MA, USA).

### Measurement of neuronal cell viability

The control or TIMP-2 plasmid transfected BV2 cells were treated with LPS for 24 h, and the conditioned media were transferred to Neuro2a cells. After 24 h of incubation, the cell viability of Neuro2a cells was checked by the 3-(4,5-dimethylthiazol-2-yl)-2,5-diphenyl tetrazolium bromide (MTT; Sigma-Aldrich) reduction assay as previously described [21].

### Statistical analysis

Unless otherwise stated, all of the experiments were performed with triplicate samples and were repeated at least three times. The data are presented as means ± the standard error of the mean (S.E.M.), and statistical comparisons between groups were performed using one-way analysis of variance followed by Newman-Keuls multiple comparison test. *P* values less than 0.05 were deemed to indicate statistical significance.

## Results

### Lipopolysaccharide inhibited tissue inhibitor of metalloproteinase-2 expression in BV2 cells and primary microglia

**Figure 1:**
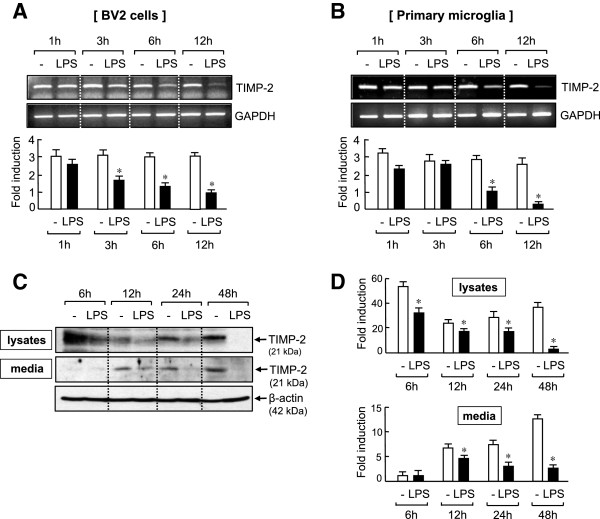
**Lipopolysaccharide (LPS) suppressed tissue inhibitor of metalloproteinase (TIMP)-2 expression in BV2 cells and primary microglia.** BV2 cells **(A)** or rat primary microglia **(B)** were treated with LPS (100 or 10 ng/ml), and total RNA was isolated at indicated times after LPS treatment. The mRNA expression of TIMP-2 was measured by RT-PCR and normalized to GAPDH expression. Representative gels are shown at the top, and quantification of three independent experiments are shown at the bottom panel. **(C,D)** Western blot analysis was performed using cell lysates or conditioned medium of BV2 cells treated with LPS (100 ng/ml). Levels of TIMP-2 protein expression were normalized using β-actin and were expressed as relative fold changes in comparison with control samples. **(C)** Representative western blots; molecular weights of TIMP-2 and β-actin are indicated. **(D)** Quantification of western blot data. Values correspond to the mean ± S.E.M. of three independent experiments. *P <0.05, significantly different from control samples.

When we measured TIMP-2 mRNA expression in LPS-treated BV2 cells at the indicated time points, TIMP-2 was constitutively expressed in resting BV2 cells, and LPS treatment significantly inhibited its expression after 3 h (Figure 1A). Conversely, in primary microglia, significant inhibition of TIMP-2 mRNA was observed after 6 h of LPS treatment, indicating some delayed regulation compared with that in the BV2 cell line (Figure 1B). To confirm the changes in TIMP-2 expression at the protein level, we performed western blot analysis in cell lysates and conditioned media collected from LPS-treated BV2 microglia. As shown in Figure 1C, TIMP-2 protein that is constitutively expressed in cells was time-dependently inhibited by LPS treatment. The basal expression of TIMP-2 was highest at 6 h and decreased thereafter, probably due to its secretion into the media. Notably, in resting microglia, TIMP-2 secretion was increased up to 48 h and was inhibited by LPS treatment (Figure 1C, D).

### Overexpression of tissue inhibitor of metalloproteinase-2 suppressed the production of pro-inflammatory cytokines, nitric oxide, and reactive oxygen species, while increasing anti-inflammatory IL-10 production

**Figure 2:**
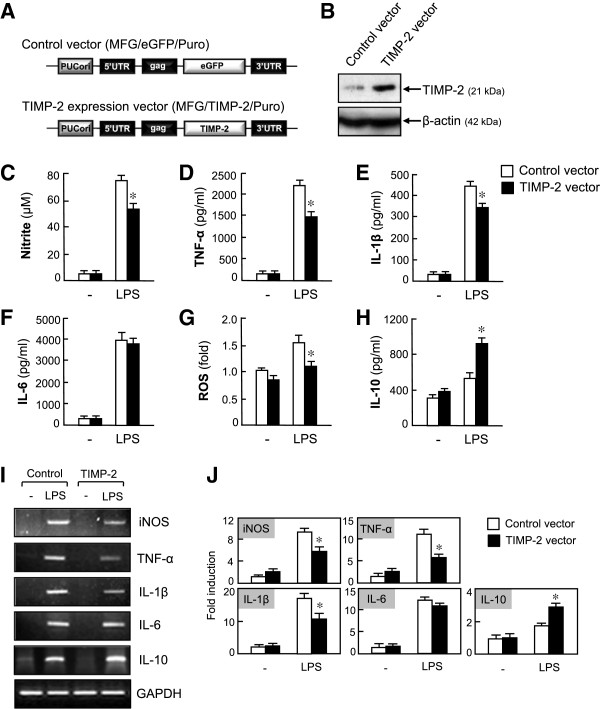
**Overexpression of tissue inhibitor of metalloproteinase (TIMP)-2 inhibited the production of nitric oxide (NO), TNF-α, IL-1β, and reactive oxygen species (ROS), while increasing anti-inflammatory IL-10 production in lipopolysaccharide (LPS)-stimulated BV2 cells. (A)** Control vector and TIMP-2 expression plasmid. **(B)** Western blot for TIMP-2 in BV2 cells transfected with TIMP-2 or control vector. **(C-H)** Effects of TIMP-2 on pro- or anti-inflammatory molecules. BV2 cells transfected with TIMP-2 or control vector were treated with LPS (100 ng/ml) for 16 h, and the amounts of NO, TNF-α, IL-1β, IL-6, and IL-10 released into the media, as well as the intracellular ROS level, were measured. **(I)** BV2 cells transfected with TIMP-2 or control vector were treated with LPS (100 ng/ml) for 6 h, and total RNA was isolated. The mRNA levels of inducible nitric oxide synthase (iNOS) and cytokines were determined by RT-PCR. **(J)** Quantification of RT-PCR data. Values correspond to the mean ± S.E.M. of three independent experiments. *P <0.05, significantly different from the LPS + control vector group.

To investigate the role of TIMP-2 in activated microglia, the TIMP-2 expression vector (Figure 2A) was introduced into BV2 cells by transient transfection. We confirmed that the expression of TIMP-2 was upregulated in TIMP-2 vector-transfected cells (Figure 2B). The overexpression of TIMP-2 reduced LPS-induced secretion of NO, TNF-α, IL-1β without affecting the secretion of IL-6 (Figure C-F). In addition, TIMP-2 reduced intracellular ROS production in LPS-stimulated microglia (Figure 2G). Conversely, TIMP-2 increased the production of the anti-inflammatory cytokine, IL-10 (Figure 2H). Likewise, RT-PCR analysis showed that TIMP-2 suppressed LPS-induced mRNA expression of iNOS, TNF-α, and IL-1β and upregulated IL-10 expression (Figure 2I, J).

### Knockdown of tissue inhibitor of metalloproteinase-2 expression by small interfering RNA augmented inflammatory responses

**Figure 3:**
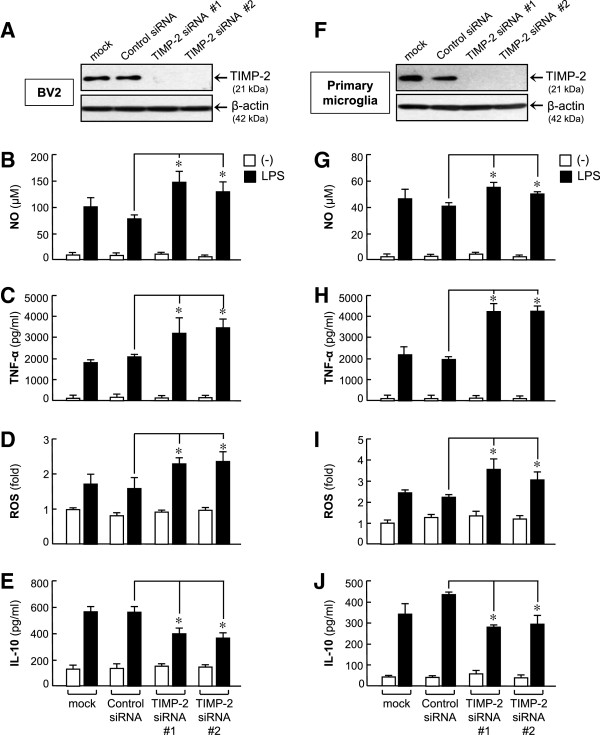
**Knockdown of tissue inhibitor of metalloproteinase (TIMP)-2 aggravated inflammatory responses. (A)** TIMP-2 protein reduction by small interfering RNAs (siRNAs) was confirmed by Western blotting. **(B-E)** Effects of TIMP-2 knockdown on nitric oxide (NO), TNF-α, reactive oxygen species (ROS), and IL-10 production in lipopolysaccharide (LPS)-stimulated BV2 cells. Cells transfected with TIMP-2 siRNA were treated with LPS (100 ng/ml) for 16 h, and the amounts of NO, TNF-α, IL-10 released into the media and intracellular ROS level were measured. **(F)** TIMP-2 knockdown in primary microglia. **(G-J)** The effects of TIMP-2 knockdown on pro-/anti-inflammatory molecules were confirmed in LPS-stimulated primary microglia. Values are expressed as the means ± S.E.M. for three replicates using different cell cultures. *P <0.05, significantly different from the LPS + control siRNA group.

To assess the effects of TIMP-2 in activated microglia, BV2 cells were transfected with two different TIMP-2 siRNAs (#1 and #2), which specifically target TIMP-2 at different binding sites (Figure 3A). Next, the cells were incubated with LPS for 16 h. We found that both siRNAs elevated NO, TNF-α, and ROS production, but inhibited IL-10 production compared with the control siRNA-treated groups (Figure 3B-E). Similar results were observed using primary microglial cultures (Figure 3F-J). The results indicate that endogenous TIMP-2 plays an anti-inflammatory role in activated microglia. We found that siRNA transfection did not significantly affect BV2 cell viability (data not shown).

### Overexpression of tissue inhibitor of metalloproteinase-2 suppressed the activity and expression of lipopolysaccharide-induced matrix metalloproteinase-3, -8, and -9

**Figure 4:**
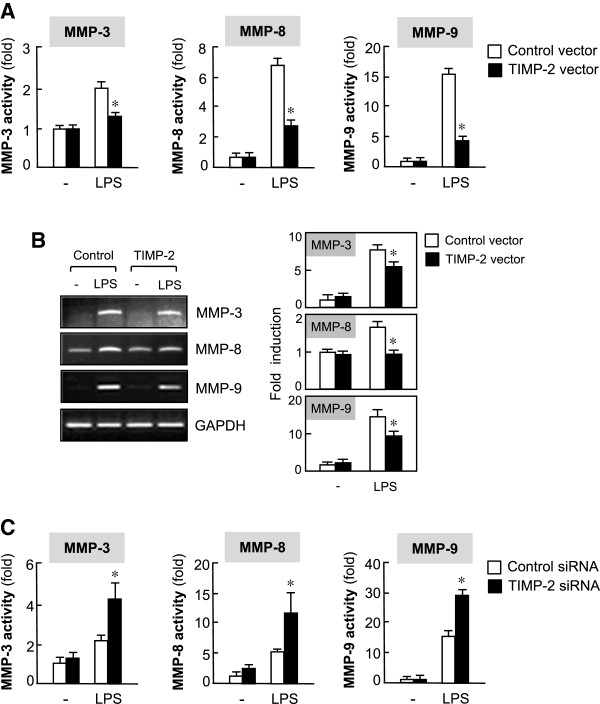
**Tissue inhibitor of metalloproteinase (TIMP)-2 suppressed the activity and expression of lipopolysaccharide (LPS)-induced matrix metalloproteinase (MMP)-3, -8 and -9. (A)** BV2 cells transfected with TIMP-2 or control vector were treated with LPS (100 ng/ml) for 16 h, and the enzymatic activity of MMPs in cell culture supernatants were assessed using MMP activity assay kits. MMP activity units were expressed as a change in fluorescence intensity. Values are expressed as the means ± S.E.M. for three independent experiments. **(B)** Cells transfected with TIMP-2 or control vector were treated with LPS (100 ng/ml) for 6 h, and the mRNA expression of MMPs was monitored by RT-PCR. Representative gels are shown in the left panel, and quantification data are shown in the right panel (n = 3). *P < 0.05, significantly different from the LPS + control vector group. **(C)** Knockdown of TIMP-2 augmented MMP activities. *P < 0.05, significantly different from the LPS + control small interfering RNA (siRNA) group.

TIMP-2 is known to be an endogenous inhibitor of MMPs. Thus, we investigated whether TIMP-2 inhibits the activity of MMP-3, -8 and -9, which have been previously reported to be important pro-inflammatory mediators in activated microglia [8,9]. As shown in Figure 4A, TIMP-2 overexpression reduced the activity of MMP-3, -8 and -9 in LPS-stimulated BV2 cells. In addition, RT-PCR analysis showed that TIMP-2 suppressed MMP-3, -8, and -9 expression at the mRNA level (Figure 4B). In contrast, knockdown of TIMP-2 elevated MMP-3, -8, and -9 activities (Figure 4C). The results suggest that TIMP-2 may exert anti-inflammatory effects through the reduction of MMPs .

### Tissue inhibitor of metalloproteinase-2 abrogated lipopolysaccharide-induced phosphorylation of three types of mitogen-activated protein kinases

**Figure 5:**
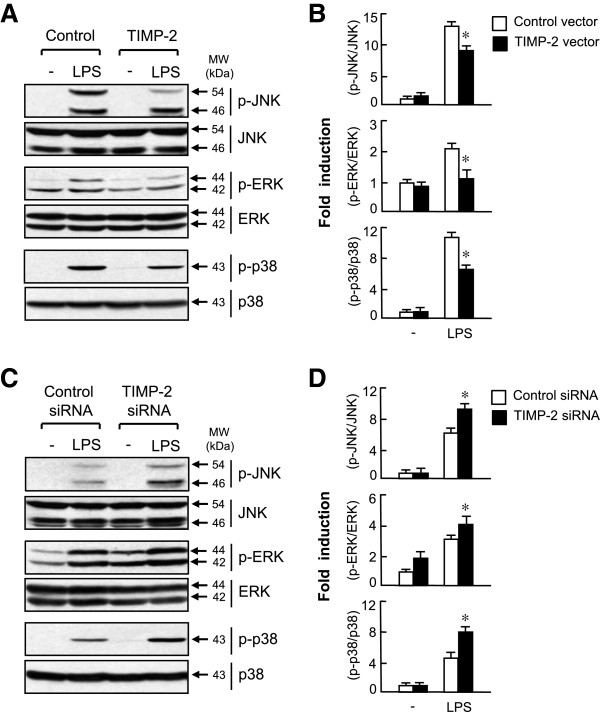
**Mitogen-activated protein kinase** (**MAPK) signaling pathways are involved in the anti-inflammatory mechanism of tissue inhibitor of metalloproteinase (TIMP)-2. (A)** Cells transfected with TIMP-2 or control vector were treated with lipopolysaccharide (LPS) (100 ng/ml) for 30 min, and were subjected to immunoblot analysis using antibodies against the phospho- or total forms of JNK, ERK, and p38. **(B)** Quantification of western blot data. Levels of the active forms of MAPKs were normalized with respect to the levels of the total form of MAPKs, and then were expressed as relative fold changes compared with the MAPK levels in control samples. *P <0.05, significantly different from the LPS + control vector group. **(C)** Knockdown of TIMP-2 augmented MAPK phosphorylation in LPS-stimulated BV2 cells. **(D)** Quantification of Western blot data. *P <0.05, significantly different from the LPS + control small interfering RNA (siRNA) group.

Next, we examined the effect of TIMP-2 on the activity of MAP kinases, which are crucial signaling molecules involved in inflammatory reactions and MMP gene expression. Western blot analysis revealed that overexpression of TIMP-2 significantly inhibited LPS-induced phosphorylation of JNK, ERK, and p38 MAPK (Figure 5 A and B). Conversely, knockdown of TIMP-2 augmented MAPK phosphorylation (Figure 5C and D). The data suggest that MAPK signaling pathways are involved in the anti-inflammatory effect of TIMP-2 in activated microglia.

### Tissue inhibitor of metalloproteinase-2 inhibited the DNA binding and transcriptional activities of NF-κB but enhanced the DNA binding and transcriptional activities of Nrf2 and cAMP-response element binding protein in lipopolysaccharide-stimulated microglia

**Figure 6:**
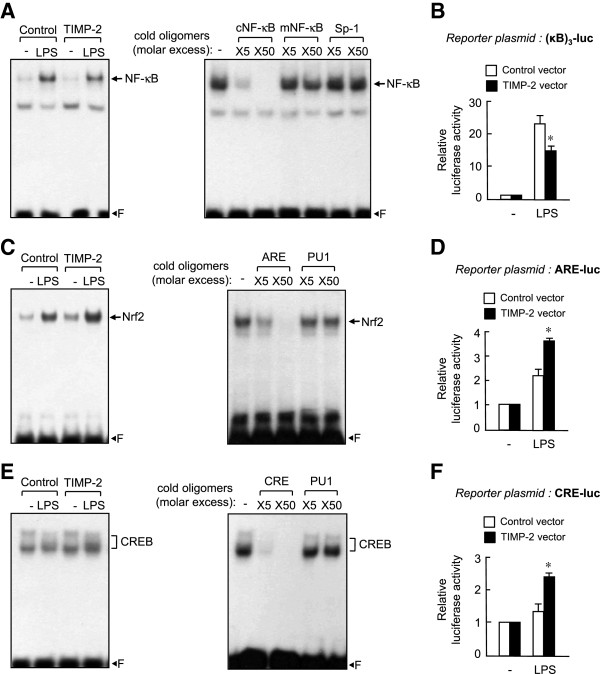
**Tissue inhibitor of metalloproteinase (TIMP)-2 suppressed the DNA binding and transcriptional activities of NF-κB but enhanced the DNA binding and transcriptional activities of Nrf2 and cAMP-response element binding protein (CREB).** Nuclear extracts were prepared from BV2 cells transfected with TIMP-2 or control vector after treatment with lipopolysaccharide (LPS) (100 ng/ml) for 3 h. Electrophoretic mobility shift assay (EMSA) was performed using NF-κB **(A)**, anti-oxidant response element (ARE) **(C)**, or cAMP response element (CRE) **(E)** probe. Competition assays indicate that the DNA-protein complex is sequence specific because the amount of complex was diminished by a molar excess of cold oligonucleotides but not by mutant (mNF-κB) or nonspecific oligonucleotides (Sp-1 or PU1). ‘F’ indicates free probe. Transcriptional activity of the reporter plasmids **(B)** (κB)_3_-luc, **(D)** ARE-luc and **(F)** CRE-luc in TIMP-2-overexpressed BV2 cells. Cells co-transfected with reporter plasmid and TIMP-2 vector were treated with LPS (100 ng/ml) for 6 h, and reporter gene assay was performed. Data are reported as the means ± S.E.M. for three separate experiments. *P <0.05, significantly different from the LPS + control vector group.

To explore the anti-inflammatory mechanism of TIMP-2, we examined the effect of TIMP-2 on NF-κB, which is an important transcription factor regulating cytokines and MMP gene expression in microglia [25]. As shown in Figure 6A, NF-κB DNA binding activity was reduced in TIMP-2 overexpressed microglia compared with that in control cells. NF-κB transcriptional activity was assayed by co-transfecting BV2 cells with the TIMP-2 expression plasmid and NF-κB reporter plasmid containing three NF-κB binding sites. The luciferase activity assay showed that overexpression of TIMP-2 inhibited NF-κB-mediated transcriptional activation (Figure 6B). Next, we examined the effect of TIMP-2 on Nrf2 and cAMP-response element binding protein (CREB), which are key transcription factors mediating anti-inflammatory and antioxidant functions in microglia [26]. We observed that TIMP-2 augmented the DNA binding and transcriptional activities of Nrf2 and CREB in LPS-stimulated microglia (Figure 6C-F). The data suggest that the NF-κB, Nrf2, and CREB pathways are largely involved in TIMP-2-mediated anti-inflammatory effects in microglia.

### Overexpression of tissue inhibitor of metalloproteinase-2 attenuated neuronal cell death induced by activated microglia

**Figure 7:**
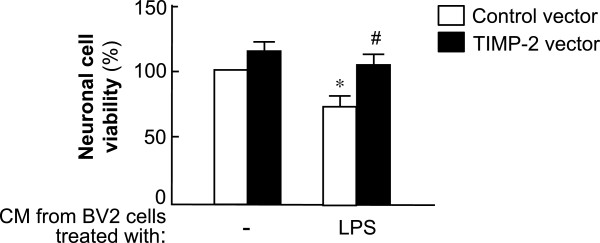
**Tissue inhibitor of metalloproteinase (TIMP)-2 attenuated neuronal cell death induced by activated microglia.** Neuro2a cells were incubated with conditioned media (CM) from lipopolysaccharide (LPS)-treated BV2 cells transfected with TIMP-2 or control vector. After 24 h of incubation, the MTT assay was performed to determine cell viability. Values are the mean ± S.E.M. of three independent experiments. *P <0.05, significantly different from non-LPS treated control cells. ^#^*P* <0.05, significantly different from the LPS + control vector group.

We examined whether TIMP-2 affects the viability of neuronal cells by modulating microglial activation. To determine the effect of soluble factors released from activated BV2 cells on Neuro2a cell viability, conditioned media from BV2 cells were transferred to Neuro2a cells. After incubation for 24 h, the cell viability of Neuro2a cells was measured using the MTT assay. We found that the cell viability of Neuro2a was markedly improved by overexpression of TIMP-2 compared with that in the control vector group (Figure 7). Interestingly, we also found that the cell viability of BV2 cells was modestly increased in the TIMP-2-transfected group (data not shown). Therefore, these data indicate that the cytoprotective effects of TIMP-2 were due to the reduced secretion of proinflammatory/neurotoxic mediators from TIMP-2-overexpressed microglia.

## Discussion

In the present study, we demonstrated the role of TIMP-2 in LPS-induced microglial activation. We found that TIMP-2 is constitutively expressed in microglia, but this expression was significantly inhibited by LPS treatment. By utilizing TIMP-2 overexpression and knockdown, the current study demonstrates the anti-inflammatory role of endogenous TIMP-2 that may be attenuated by immunostimulatory conditions. We showed that overexpression of TIMP-2 suppresses the production of pro-inflammatory molecules, whereas TIMP-2 knockdown augmented them. Moreover, TIMP-2 inhibits three types of MMPs (MMP-3, -8 and -9) and MAPK/NF-κB signaling pathways. Lastly, TIMP-2 overexpression afforded neuroprotection via reduced microglial activation. Therefore, TIMP-2 is thought to be a crucial regulator of neuroinflammation.

Several studies have reported the role of TIMP-2 in the CNS. TIMP-2 showed neuroprotective effects in an animal model of stroke by reducing the proteolytic opening of the blood-brain barrier and subsequent intracerebral hemorrhage [27,28]. Along with these findings, transplantation of autologous bone marrow cells overexpressing TIMP-2 reduced ischemic damage [29]. TIMP-2 was significantly increased in the serum or cerebrospinal fluid (CSF) of stroke, MS, AD, or HD patients, suggesting the protective role of TIMP-2 in neurological disorders [15-17]. Furthermore, TIMP-2 deficient mice demonstrated motor dysfunction, including trembling prior to locomotion, excessive jumping upon moving, implying the involvement of TIMP-2 in developmental and behavioral alterations [30]. Because microglial activation is associated with neuropathological conditions, the inhibition of microglial activation by TIMP-2 may be one factor contributing to TIMP-2-mediated neuroprotection.

Our group recently reported that LPS increased the expression of MMP-3, -8, and -9 in microglia [8]. Specific inhibition of these MMPs strongly suppressed inflammatory reactions induced by LPS. Thus, inhibition of MMP-3, -8, or -9 inhibited the expression of iNOS and proinflammatory cytokines and also suppressed MAPK and NF-κB activities, as well as ROS production [8]. We demonstrated that MMP-3, -8, -9 play pro-inflammatory roles by activating protease activated receptor-1 on the surface of microglia and/or by cleavage/activation of proTNF-α [9]. In the present study, we found that TIMP-2 overexpression suppressed proinflammatory signaling in a manner similar to that of MMP inhibitors. The data suggest that the anti-inflammatory effect of TIMP-2 is the result of a mechanism dependent on the MMP pathway.

Activated microglia release inflammatory cytokines and neurotoxic factors, as well as amplify the inflammatory response in an autocrine or paracrine manner. Additionally, released toxic factors from activated microglia cause neuronal death, which contributes to neurodegeneration in a positive feedback loop [3-6]. Our findings suggest that the neuroprotective effects of TIMP-2 were due to the reduced secretion of proinflammatory/neurotoxic mediators from microglia. Thus, TIMP-2 may likely control neurotoxicity through the modulation of microglial activation.

## Conclusions

The present study demonstrated that TIMP-2 exerts anti-inflammatory effects through the reduction of MMPs and pro-inflammatory molecules. Therefore, the endogenous system of TIMP-2, which can modulate the abnormal expression of MMPs and the concomitant inflammatory response, may be a promising target for the treatment of various neuroinflammatory disorders.

## Abbreviations

AD: Alzheimer’s disease; ARE: anti-oxidant response element; CM: conditioned media; CRE: cAMP response element; CREB: cAMP-response element binding protein; EMSA: electrophoretic mobility shift assay; ERK: extracellular signal-regulated kinase; HD: Huntington’s disease; iNOS: inducible nitric oxide synthase; JNK: c-Jun N-terminal kinase; LPS: lipopolysaccharide; MAPK: mitogen-activated protein kinase; MMP: matrix metalloproteinase; MS: multiple sclerosis; NF-κB: nuclear factor-κB; Nrf: NF-E2-related factor; siRNA: small interfering RNA; ROS: reactive oxygen species; TIMP: tissue inhibitor of metalloproteinase.

## Competing interests

The authors declare that they have no competing interests.

## Authors’ contributions

EL designed the study and performed the experiments and wrote the manuscript. HK supervised the design of the study and analyzed the data and wrote the manuscript. Both authors read and approved the final manuscript.
